# Family and Individual Risk and Protective Factors of Depression among Chinese Migrant Children with Oppositional Defiant Disorder Symptoms

**DOI:** 10.3389/fpsyg.2017.00508

**Published:** 2017-04-04

**Authors:** Xu Liu, Xiuyun Lin, Qing Zhou, Nan Zhou, Yanbin Li, Danhua Lin

**Affiliations:** ^1^Institute of Developmental Psychology, Beijing Normal UniversityBeijing, China; ^2^Department of Psychology, University of CaliforniaBerkeley, CA, USA

**Keywords:** migrant children, risk factors, protective factors, family factors, individual factors, oppositional defiant disorder symptoms

## Abstract

Migrant children reached 35.81 million in China and were vulnerable to serious emotional problems including depression. The present study aimed to identify the family and individual risk and protective factors for depression in an at-risk sample of Chinese migrant children. Participants were 368 children (9.47 ± 1.46 years old, 73.4% boys) who had at least one symptom of Oppositional Defiant Disorder symptoms (ODD) and their parents in Mainland China. Risk and protective factors within both family (i.e., family maltreatment and family functioning) and individual (i.e., automatic thoughts and resilience) perspectives. Family maltreatment and negative automatic thoughts served as risk factors in relation to children's depression. Further, automatic thoughts mediated the relationship between family maltreatment and children's depression. Family functioning (cohesion, but bot adaptability) and individual resilience could buffer the effects of risk factors in the Structure Emotion Model such that both cohesion and resilience moderated the relationship between family maltreatment and children's automatic thoughts only. Our findings highlighted the urgent need to decrease risk factors and increase protective factors of both family and child individual characteristics in prevention and intervention depression among migrant children with ODD symptoms in China.

## Introduction

Migration and immigration is certainly becoming one of the great issues all around the world (Dogra et al., 2011; Crosnoe and Fuligni, 2012). With the rapid development of economy and urbanization in China, as of 2014, there were over 35.81 million children (or 12.84% of all children) aged from 0 to 17 who migrated from rural to urban areas in China. In urbanized areas such as Beijing, it was estimated that as high as 36.28% of children were migrants (NWCCW et al., 2014). A growing body of research suggested that migration was a complex and stressful process particular for children and adolescents, and had been associated with elevated risks for emotional (e.g., depression, anxious, loneliness), behavioral (e.g., aggression, withdrawal, delinquency), and other adjustment problems (Jordan and Graham, 2012; Washbrook et al., 2012). Oppositional defiant disorder (ODD) was a collection of emotional and behavioral problems, as children with ODD were characterized by a pattern of moody/irritable, argumentative/defiant, and hostile/vindictive behavior toward authority figures (American Psychiatric Association, 2013). Moreover, ODD symptoms were identified to be associated with more life stress (Lavigne et al., 2012). Targeting on children with ODD symptoms could be meaningful to understand those migrant children with emotional and behavioral problems.

## Depression in Chinese migrant children with odd symptoms

Given the critical role of emotional wellbeing in children's academic development and social relationship, particularly in clinic or at risk populations (Carlson and Cantwell, 1980; Izard et al., 2001), study on depression among migrant children with ODD symptoms can help to a further understanding of depression and inform treatment to children with ODD (Latimer et al., 2012). Indeed, there were some preliminary empirical evidences that migrant children in China did have emotional problems, such as depression (Yuan et al., 2012; Hu et al., 2014). Migrant children in China had more severe depression than local urban children (Zeng and Li, 2007), and even scored significantly higher than general rural children on depression (Lin et al., 2009). Moreover, childhood ODD was associated with an increased risk of depression (Hazell, 2010). It is essential to a better understanding of depression among migrant children with ODD symptoms.

## Risk and protective factors for depression among migrant children

For risk factors relation to migrant children and children with ODD symptoms, child maltreatment executed by parents and children individual negative automatic thought were not be ignored. Parents of migrant children were more inclined to use harsh and abusive upbringing, particularly with child with ODD (Li et al., 2016b), as migrant families tended to have lower socio-economic status (SES) and less educated parents compared to non-migrant families (Li et al., 2008; Hou et al., 2009). Due to defiant problems and ill temper of children with ODD symptoms, they had an increased risk of being maltreated by their parents and caregivers (Gershoff, 2002; Lin et al., 2014). Research that focused on Chinese children with ODD indicated that parents often emotionally punished (e.g., shaming, blaming, and isolating) and/or physically punished their children (e.g., kicking, beating, slapping, hitting the child's buttocks, and twisting an ear; Lin et al., 2014). And this, consequently, was bound to increase children's depression (Li et al., 2016a). Moreover, the process of migration might be a very potent trigger for children's automatic thoughts, for migration always accompanied by a series of negative life events such as separation from friends and other family members, economic stressors, and perceived discrimination (Fang et al., 2008; Lin et al., 2009). Subsequently, those children with more negative automatic thoughts were vulnerable to have higher levels of depression (Clarke and Goosen, 2009; Hjemdal et al., 2013; Du et al., 2015). Additionally, cognitive theory (Beck, 1979) asserted that negative cognition interpretations of experience lead to negative views of self, world, and future, developed automatic and affected feelings and behavior, and further leaded to the emotional problems.

Despite exposure to risk factors or under adversities, more than half of children who experienced early adversity grew into competent, confident and caring individuals (Kashani et al., 1995; Wingo et al., 2010). And these differences may depend on both their families' circumstances (Kashani et al., 1995) and characteristics of children themselves (Hjemdal et al., 2006; Alim et al., 2008; Wingo et al., 2010). Optimal family functioning and higher level of personal resilience were always identified as family and individual protective factors for children developing into psychopathology.

Children who with a better family functioning usually had less emotional problems, and so they behaved well accordingly (Tamplin and Gooyer, 2001; Fang et al., 2004). Olson et al. (1979) developed the Circumplex Model and placed family functioning into adaptability and cohesion dimensions. Specifically, adaptability assessed the family ability to change in response to situational stress and cohesion meant the degree of emotional bonding between family members. Olson et al. (1979) studies showed the optimal family functioning was associated with moderate levels of cohesion and adaptability. Some studies showed that both adaptability and cohesion were negatively associated with children's depression (Cumsille and Epstein, 1994; Xu et al., 2008), however, some demonstrated that cohesion, but not adaptability, was negatively associated with children's depression (Kashani et al., 1995; Li et al., 2008). Moreover, studies has proved family functioning could be a moderator to the mental healthy, especially for those in adversity (Robbins et al., 2003). However, considering almost every migrant child has once stayed at home as left-behind child, they may have a less intimate or less close relationship with their parents (Duan, 2015). Consequently, family functioning in migrant family may be less cohesive and less adaptable. Whether the less cohesive and the less adaptable family functioning could be a buffer for depression with migrant children particular those with ODD symptoms? To a comprehensive consideration, we still propose both family adaptability and family cohesion could mitigate the threat of family maltreatment in current study.

As for individual protective factor, resilience has been identified as an important factor in the generation and the prevention of depression according to a series studies (Hjemdal et al., 2006; Alim et al., 2008). Rutter (2006) have proposed that resilience starts with a recognition of the huge individual variation responses to risk experiences, and further have applied for intervention strategies with respect to prevention. Later, three approaches on resilience's application, including the harm-reduction, the protection, and the promotion approach, were developed (Davydov et al., 2010). According to the protection approach, resilience has been viewed as a defense mechanism (analogous to “immune barriers”) to preserve health and protect against the negative outcomes occurring when confronted adversities (Patel and Goodman, 2007). Converse to children with less resilience, children with high level of resilience were capable to positively comprehend the negative life events rather than been frustrated. Moreover, these children utilized their positive attitude and ways to solve problems (Shannon et al., 2007; Davydov et al., 2010; Wang et al., 2014). Therefore, children with higher resilience were able to reduce the negative impact of the adversities and develop into health outcomes, which assist them well-adapted. Specifically, Wingo et al. (2010) have demonstrated that resilience as a main moderator mitigated the severity of depressive symptom in individuals exposed to childhood abuse or other traumas. Wang and Lin (2012) and Wang et al. (2014) also have found among Chinese migrant children that these children's high level of resilience reduced their emotional problems and promoted their adaption to the new environment. Although, resilience was confirmed as a moderator during stress or trauma, less was known for its work-effectively process. Did resilience kick in when exposing to the life stress directly? Or it was solely worked effectively in the person-internal systems involving cognition? Moreover, did resilience play an equally important role in reducing adverse impact for high-risk migrant children, such as migrant children with ODD? In the present study, we assume children's resilience play as moderators in the beginning of facing family maltreatment as well as the processes of developing automatic thoughts and depression, even for migrant children with ODD symptoms.

## The complex relationship of risk and protective factors and children's depression

A review by Grant et al. (2006) indicated that familial and individual factors were associated with children's psychopathology symptoms in complex processes and there always existed moderators or mediators. However, less was known for the process that how risk (i.e., maltreatment, negative automatic thoughts) and protective factors (i.e., family functioning and resilience) of both familial and individual impact on children's depression, particularly among migrant children with ODD symptoms. According to the Family System Theory (Cox and Paley, 2003), family was a dynamic and interactive system with interdependent factors at multiple levels that factors at the whole family level and the individual level played important roles in shaping and influencing the development of a child. Identifying the familial and individual risk and protective factors for depression of Chinese migrant children with ODD symptoms is a critical step to inform the development of intervention programs that addressing the needs of this growing population.

Among these risk and protective factors we aforementioned, automatic thoughts always played as a mediator in the link between stressors (e.g., maltreatment) and depressive symptoms, as cognitive behavioral theory postulated that automatic thoughts are concomitant with a stressful situation leading to depression (Beck, 1979; Kanter et al., 2004). And empirical studies suggested that negative automatic thought was not merely an con-commitent of depression but also was a predictor of the course of depression following the family maltreatment (Hjemdal et al., 2013; Du et al., 2015). Regarding the moderators, protective factors in both family and individual (such as family functioning and individual resilience) had been examined to decrease the negative impact of risk factors. Family functioning has been tested for buffering effects and was found a strong evidence to a buffer (Robbins et al., 2003; Grant et al., 2006). Likewise, resilience moderated depressive symptom in individuals exposed to adversities in both cross-sectional and longitudinal researches (Alim et al., 2008; Wingo et al., 2010; Zhu et al., 2015).

## The present study

**Figure 1:**
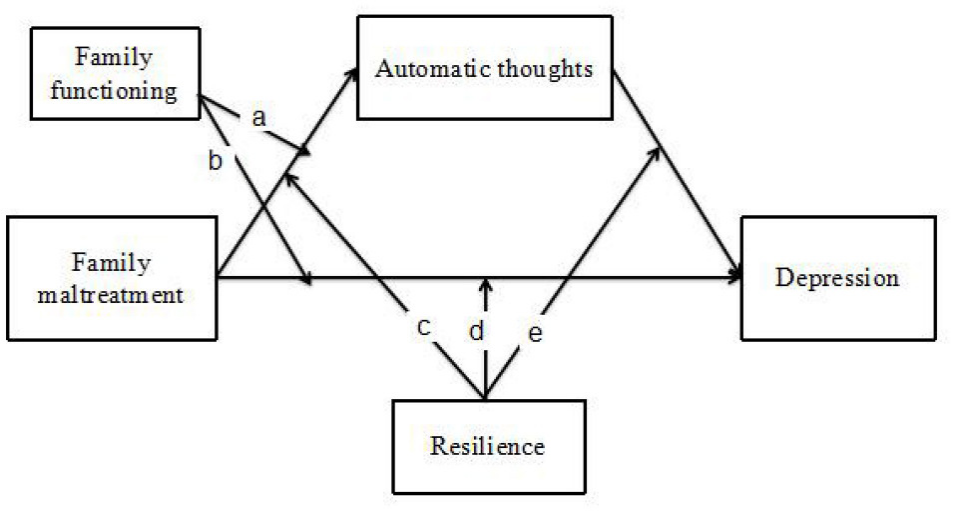
**Proposed model of risk and protective factors of both family and individual associated with depression among migration child with ODD symptoms**.

This study aimed to examine how risk (i.e., family maltreatment, automatic thoughts) and protective (i.e., family functioning, resilience) factors in both the family and the child are jointly and interactively associated with depressive symptoms in migrant children with ODD symptoms in China. We hypothesized: (1) risk factors of both family and individual would be associated with depression of migration child with ODD symptoms; (2) children's automatic thoughts would mediate the relation between family maltreatment and children's depression; (3) protective factors of both familial and individual (family functioning and child resilience) would buffer the associations between risk factors to depressive symptoms. Specifically, we hypothesized that family functioning would only moderate path a and b [i.e., the relation between family maltreatment and children's automatic thoughts (a), and the relation between family maltreatment and children's depression (b)]. By contrast, we hypothesized that child resilience would moderate three paths, including the relation between family maltreatment and children's automatic thoughts (c), the relation between family maltreatment and children's depression (d), and the relation between children's automatic thoughts and depression (e) (See Figure 1).

## Methods

### Participants and procedure

Data in the present study were drawn from a larger study conducted on migrant children in Beijing recruited from 10 elementary schools (in which 7 public elementary schools and 3 schools especially for migrant children) during 2013 and 2014. Having or not having Beijing hukou (citizenship) divided them into migrant children or Beijing urban children. We reached 10 elementary schools' school psychologists inviting them to attend the study. After getting their approval, these school psychologists assisted to deliver our research invitation letter (including a study introduction and an informed consent) to all the class master teachers to acquire their agreement to participate the research. After class master teachers agreed and signed informed consent, they were asked to nominate the children who might have ODD symptoms using ODD symptoms assessment sheet (from Diagnostic and Statistical Manual of Mental Disorders, DSM-IV). Following, a school psychological teachers, a clinical psychologist researcher together with the class master teacher further confirmed whether the selected children have ODD symptoms or not. At last, there were 375 migrant children confirmed with ODD symptoms (excluding those with intellectual disability and other disorders). Noted that we matched the contrast group (both to the non-symptoms children and to the non-migrant children), but omitted here. Next, class master teachers connected with the selected 375 children's parents and delivered an invitation of the study and two informed consent of child and parent to sign. With the exception of a refuse-participate family, we measured other 374 families that agreed to participate, including the children, their parents and their class master teachers. The teachers completed teachers' questionnaire in their office. All the selected children used the recess time in a given room in school completed the questionnaire and took their parents' questionnaire back home. After their parents completed (told to be finished by one parent in a week) the questionnaire, children brought it back to their class master teachers. All the participants were given a thank-you gift.

In conclusion, 369 questionnaires were returned to our lab. Deleting one with too many missing value, there were 368 children and parents in our study, including 270 (73.4%) boys and 98 (26.6%) girls, 154 (41.8%) father- child dyads and 188 mother- child dyads (51.1%) and 26 (7.1%) parental participants missing their gender. There were 103, 87, 102, 75 children from grade two, three, four, five, respectively, aging from 7 to 14 with the average age 9.47 ± 1.46 years old. The average migrant time of migrant children was 75.16 ± 37.85 months, in which the maximum time was 156 months and the minimum migrant time was 6 months.

### Measures

#### ODD symptoms assessment

Based on the 8 symptoms indicated in DSM-IV (American Psychiatric Association, 2013) for the diagnosis of ODD measured on a dichotomous scale (“0 = no,” “1 = yes”) (e.g., “often loses temper”; “often argues with adults”), the class master teacher, a school psychological teachers and a clinical psychologist evaluated whether a child had the ODD symptoms. As long as one had one or more than one item of the 8-item scale, child was identified with ODD symptoms. Scores from these eight items were summed with higher total scores indicating more symptoms on ODD. In current study, the Cronbach α of the scale was 0.85.

#### Family maltreatment (child reported)

We used Childhood Trauma Questionnaire (CTQ-SF, Bernstein et al., 1997), the Chinese version (Zhao et al., 2005) to measure the family maltreatment. It is a 28-items scale (including 3 items used to evaluate the validity) manifested in five dimensions: Emotional abuse (e.g., “People in my family said hurtful or insulting things to me”), Physical abuse (e.g., “People in my family hit me so hard that it left me with bruises or marks”), Sexual abuse (e.g., “Someone molested me”), Emotional neglect (e.g., “My family was a source of strength and support”), Physical neglect (e.g., “I knew that there was someone to take care of me and protect me”) and each sub-scale has 5 items. We used four CTQ sub-scales except sexual abuse one in the present study. Items with response options rated on a 5-point Likert scale ranging from “1 = Never” to “5 = Very often.” The Cronbach α were high for of the total scale (0.84) and for each subscales (0.69~0.82) in current study.

#### Family functioning (parent reported)

Family functioning were measured using the Chinese version Family Adaptability and Cohesion Evaluation Scale (FACES-III; Olson, 2000; Xu et al., 2008). With 30 items, it evaluated the family functioning on adaptability (14 items; e.g., “Family members discuss problems and feel good about the solutions.”) and cohesion dimensions(16 items; e.g., “Our family does things together”). Each participating parent assessed their perception of the actual condition in the family, using a 5-point scale (“1 = almost never” to “5 = almost always”). The Cronbach α for FACES-II were 0.84 (adaptability = 0.79 and cohesion = 0.81) in current study.

#### Resilience (children reported)

The Resilience Scale for Chinese Adolescences (Hu and Gan, 2008) was used to measure children's resilience. In which resilience was defined as individual's healthy and constructive adjustment after they had experienced serious, traumatic, or catastrophic events. It contained 27 items with dividing into 5 dimensions on a 5-point scale (“1 = never” to “5 = always”). The 5 dimensions were target focus (5 items; e.g., “I do set goals to push myself move forward.”), emotional control (6 items; e.g., “I am capable of adjusting my mood well in a short time.”), positive perception (4 items; e.g., “Adversity is an opportunity to the growth sometimes.”), family support (6 items; e.g., “My parents always encourage me to go all out.”) and interpersonal support (6 items; e.g., “I have a friend of my age, and I share my difficulties with he/she.”). Of these 5 dimensions, the first three dimensions could be seen as personal strength, and the last two dimensions as supportive strength. We used both personal strength and supportive strength in present study. The Cronbach α were high for the overall scale (0.82) and for the two subscales (personal strength = 0.77, supportive strength = 0.66) in current study.

#### Automatic thoughts (children reported)

The Children's Automatic Thoughts Scale (CATS; Schniering and Rapee, 2002) was used to measure children's automatic thoughts. All 40 items loaded onto four separate sub-scales corresponding to physical threat (10 items; e.g., “I'm going to have an accident.”), social threat (10 items; e.g., “I'm afraid of what other kids will think of me”), personal failure (10 items; e.g., “I've made such a mess of my life.”), and hostility (10 items; e.g., “If someone hurts me, I have the right to hurt them back.”). Children were asked to rate the frequency they have experienced over the past week on a 5-point scale ranging from “not at all (0)” to “all the time (4).” Total scores higher reflected a greater frequency of negative automatic thoughts. The Cronbach α were very high for of the total scale (0.96) and for each subscales (0.82~0.88) in current study.

#### Depressive symptoms (children reported)

Children's depressive symptoms were measured using the Chinese version of the Center for Epidemiological Studies Depression scale (CES-D; Radloff, 1977). It contained 20 items (e.g., “I was bothered by things that usually don't bother me.”) rated on a 4-point scale from “1 = never” to “4 = always.” Higher summed scores of 20 items indicated stronger feeling of depression. The Cronbach's α of this scale was 0.86.

### Statistical analyses

First, we used SPSS 16.0 to conduct preliminary statistical analysis. Descriptive statistics (mean and standard deviations) were calculated for all indicator variables (i.e., family maltreatment, family functioning, children's automatic thoughts, resilience, and depression) of demographic difference. Then, Pearson correlation analysis was conducted to examine the strength of associations among family maltreatment, family functioning, children's resilience, children's automatic thoughts and children's depression. Second, the proposed model of risk and protective factors of both family and individual associated with depression was conducted in Mplus 7.0 (Muthén and Muthén, 2012) to test the mediating effect of automatic thoughts and the moderating effect of family functioning and children's resilience on the relationship between family maltreatment and children's depression. Bias-corrected 95% confidence intervals for path estimates were generated via bootstrapping with 5,000 iterations (MacKinnon et al., 2002).

## Results

Descriptive statistics and correlation coefficients for study variables were given in Table 1. There were no significant gender differences between all variables. All of the bivariate correlations were statistically significant except family functioning with children's automatic thoughts and depression (*ps* > 0.05). Risk factors (family maltreatment and automatic) were positively correlated with depression and negatively correlated with protective factors (family functioning and resilience).

**Table 1:** **Descriptive statistics for study variables**.

	**1**	**1.1**	**1.2**	**1.3**	**1.4**	**2**	**2.1**	**2.2**	**3**	**3.1**	**3.2**	**4**	**4.1**	**4.2**	**4.3**	**4.4**	**5**
1 Family maltreatment	1																
1.1 Emotional abuse	0.75^***^	1															
1.2 Physical abuse	0.66^***^	0.57^***^	1														
1.3 Emotional neglect	0.72^***^	0.28^***^	0.11^*^	1													
1.4 Physical neglect	0.73^***^	0.33^***^	0.30^***^	0.52^***^	1												
2 Family functioning	−0.13^*^	–	–	–	–	1											
2.1 Cohesion	−0.15^**^	−0.12^*^	−0.04	−0.13^*^	−0.12^*^	0.93^***^	1										
2.2 Adaptability	−0.09	−0.08	0.01	−0.09	−0.10	0.94^***^	0.75^***^	1									
3 Resilience	−0.46^***^	–	–	–	–	0.11^*^	–	–	1								
3.1 Personal strength	−0.38^***^	−0.22^***^	−0.18^**^	−0.36^***^	−0.33^***^	0.11^*^	0.14^**^	0.07	0.92^***^	1							
3.2 Supportive strength	−0.43^***^	−0.34^***^	−0.24^***^	−0.31^***^	−0.34^***^	0.06	0.12^*^	0.01	0.75^***^	0.45^***^	1						
4 Automatic thoughts	0.49^***^	–	–	–	–	−0.09	–	–	−0.36^***^	–	–	1					
4.1 Personal failure	0.49^***^	0.49^***^	0.39^***^	0.22^***^	0.35^***^	−0.16^*^	−0.17^**^	0.01	−0.38^***^	−0.29^***^	−0.43^***^	0.93^***^	1				
4.2 Social threat	0.45^***^	0.49^***^	0.43^***^	0.16^**^	0.25^***^	−0.11^*^	−0.25^***^	−0.04	−0.36^***^	−0.28^***^	−0.40^***^	0.93^***^	0.84^***^	1			
4.3 Physical threat	0.45^***^	0.46^***^	0.44^***^	0.14^**^	0.30^***^	−0.06	−0.10^*^	−0.01	−0.32^***^	−0.24^***^	−0.40^***^	0.94^***^	0.85^***^	0.82^***^	1		
4.4 Hostility	0.39^***^	0.42^***^	0.40^***^	0.10	0.25^***^	−0.06	−0.11	−0.01	−0.28^**^	−0.23^***^	−0.31^***^	0.89^***^	0.72^***^	0.75^***^	0.78^***^	1	
5 Depression	0.48^***^	–	–	–	–	−0.07	–	–	−0.51^***^	–	–	0.74^***^	–	–	–	–	1
Total mean (SD)	10.95	1.76	1.69	2.41	1.96	3.47	3.60	3.33	3.43	3.51	3.30	0.77	0.72	0.72	0.66	0.98	1.84
(*N* = 368)	(0.62)	‘(0.86)	(0.80)	(1.08)	(0.73)	(0.54)	(0.57)	(0.58)	(0.55)	(0.65)	(0.62)	(0.72)	(0.77)	(0.80)	(0.73)	(0.81)	(0.52)
Boys mean (SD)	1.95	1.72	1.70	2.40	1.98	3.48	3.60	3.35	3.43	3.51	3.30	0.75	0.70	0.69	0.64	0.96	1.84
(*N* = 270)	(0.61)	(0.82)	(0.80)	(1.10)	(0.74)	(0.55)	(0.58)	(0.58)	(0.54)	(0.64)	(0.60)	(0.71)	(0.76)	(0.79)	(0.73)	(0.81)	(0.50)
Girls mean (SD)	1.95	1.84	1.65	2.44	1.89	3.44	3.60	3.28	3.44	3.52	3.29	0.82	0.76	0.81	0.70	1.02	1.85
(*N* = 98)	(0.66)	(0.96)	(0.81)	(1.01)	(0.70)	(0.53)	(0.56)	(0.58)	(0.59)	(0.69)	(0.66)	(0.73)	(0.80)	(0.83)	(0.74)	(0.82)	(0.57)

*** *Correlation is significant at the 0.001 level (2-tailed)*.

** *Correlation is significant at the 0.01 level (2-tailed)*.

* *Correlation is significant at the 0.05 level (2-tailed)*.

**Figure 2:**
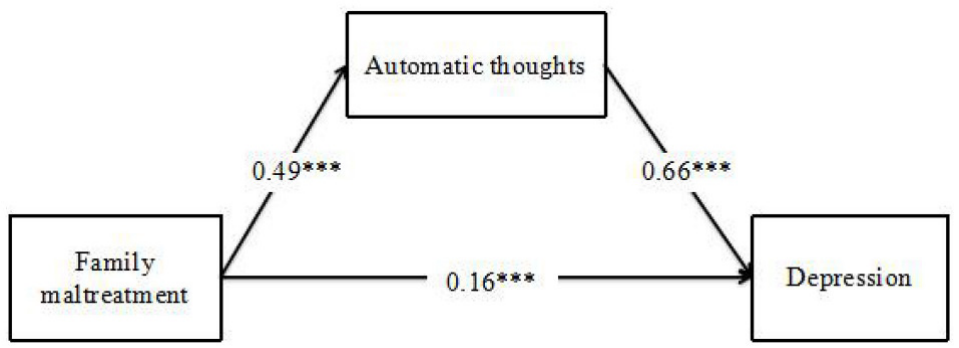
**The Mediation Model of Family maltreatment, Automatic thoughts, and Depression**. ^***^p < 0.001.

To test the mediation hypothesis, a path analysis was used to examine whether children's automatic thoughts mediated the relationship between family maltreatment and their depression (See Figure 2). The mediation model fit presented a good fit (CFI = 1.00, TLI = 1.00, RMSEA = 0.00, 95% CI = [0.26, 0.38]).

**Figure 3:**
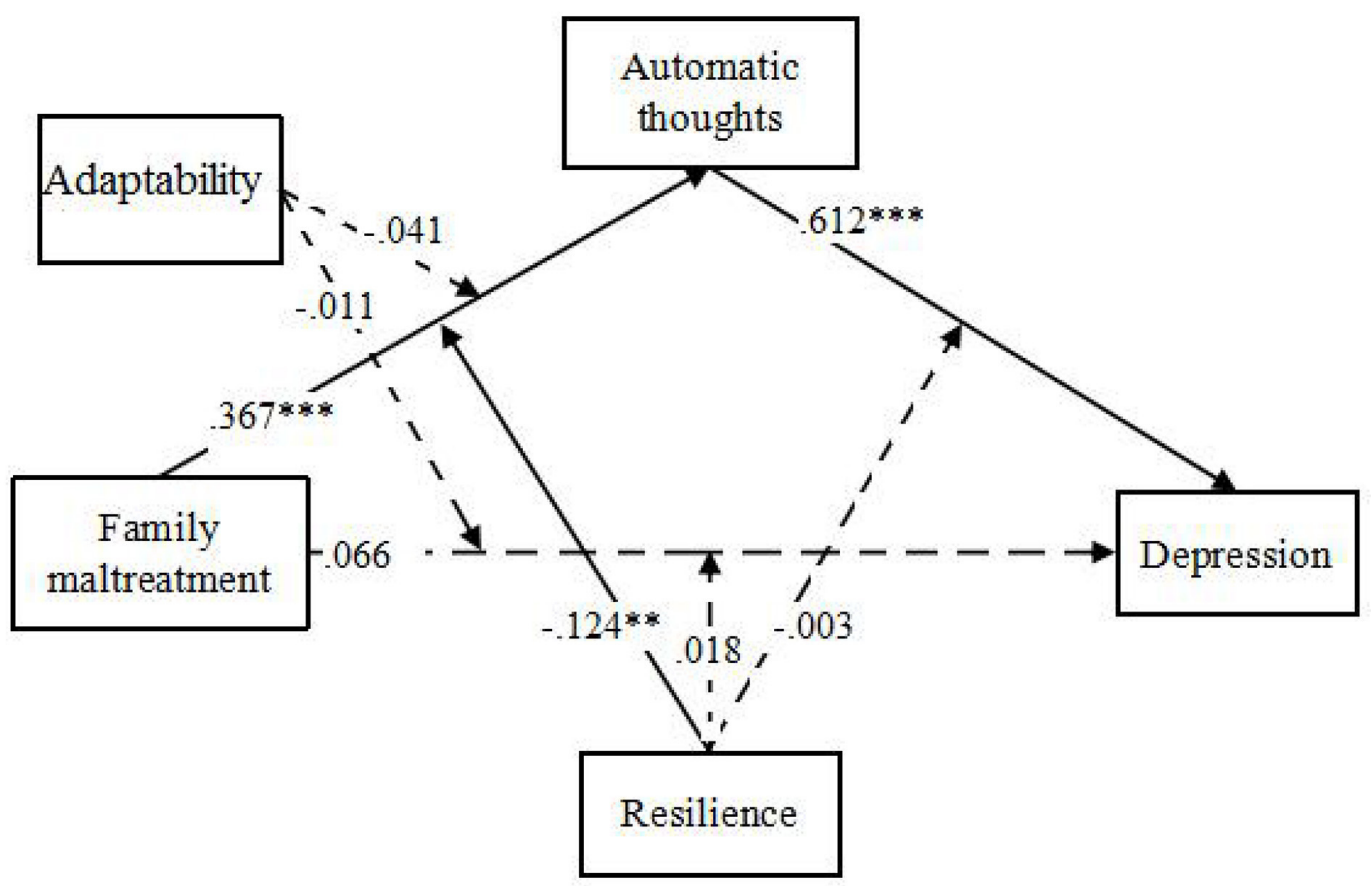
**The Moderated Mediation Model with Adaptability and Resilience**. ^**^p < 0.01, ^***^p < 0.001.

**Figure 4:**
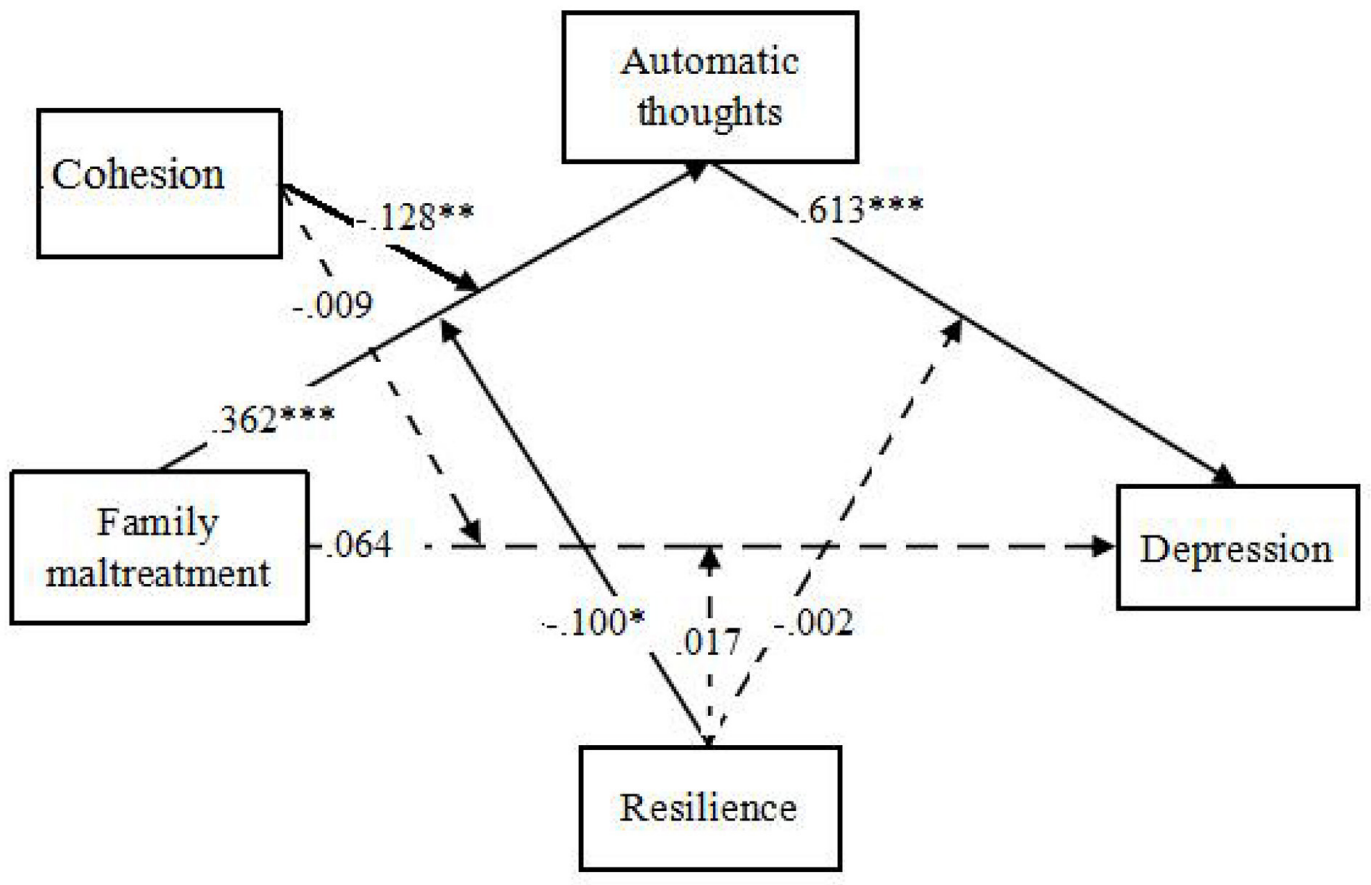
**The Moderated Mediation Model with Cohesion and Resilience**. ^*^p < 0.05, ^**^p < 0.01, ^***^p < 0.001.

We then tested the moderated mediation model by adding the two hypothesized moderators, family functioning (adaptability and cohesion) and resilience with 5 moderated paths according proposed model in Figure 1. Considering adaptability and cohesion could be different moderators as aforementioned, 2 moderated mediation models were tested separately following. The moderated mediation model with adaptability and resilience (See Figure 3) terminated normally and the model fit the data well (χ^2^ = 60.85, *df* = 1, CFI = 0.90, RMSEA = 0.05). It showed that only resilience, but not adaptability, could be a moderator. And resilience could only moderate the relationship between family maltreatment and automatic thoughts (path c). The moderated mediation model with cohesion and resilience (See Figure 4) terminated normally and the model fit the data well (χ^2^ = 55.50, *df* = 1, CFI = 0.90, RMSEA = 0.05). It showed that both cohesion and resilience could be moderators. Both of them moderated the relationship between family maltreatment and automatic thoughts (path a and path c).

**Figure 5:**
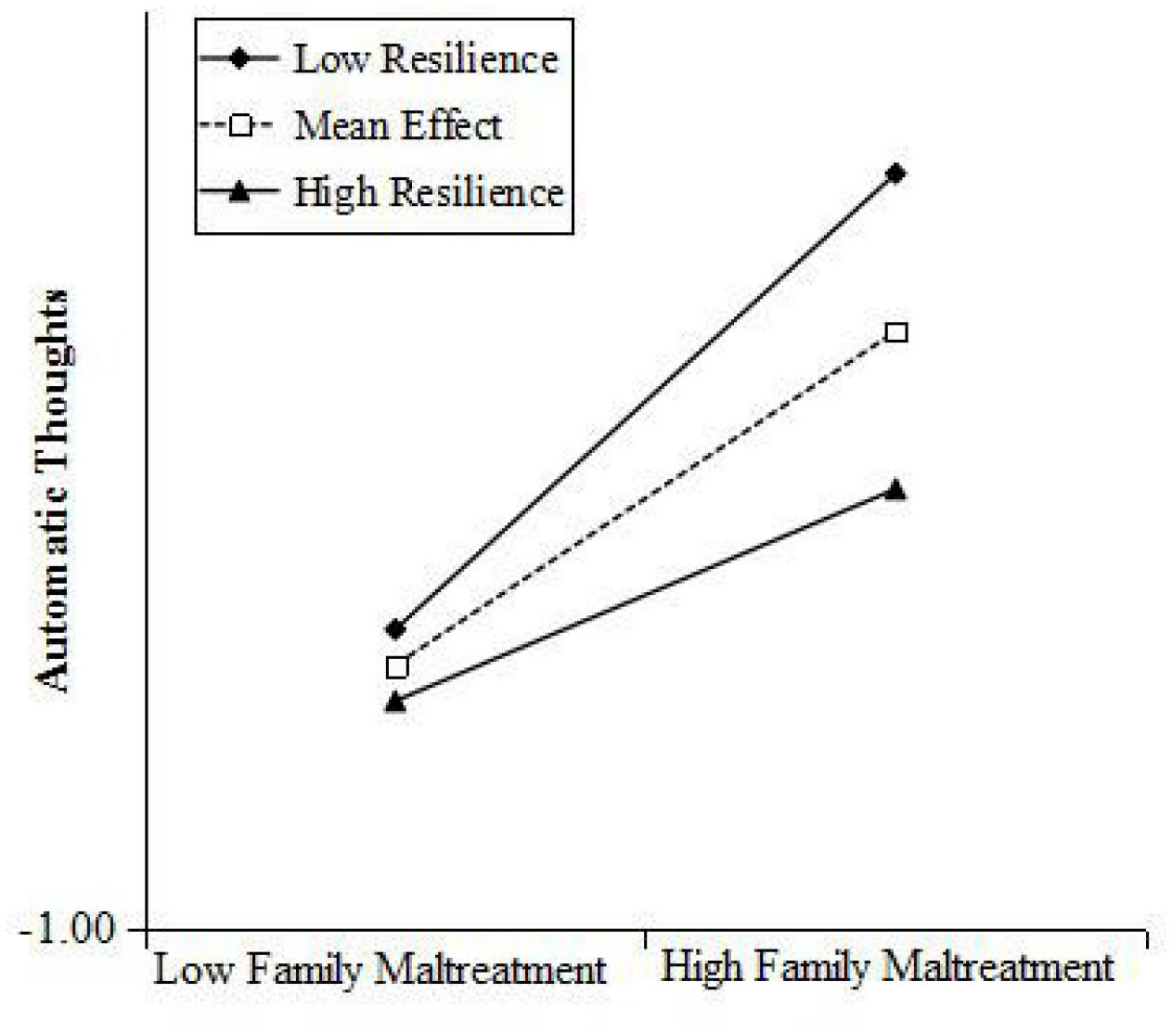
**Resilience moderated the relationship between family maltreatment and their automatic thoughts: the risk effects of family maltreatment to their automatic thoughts decreased significantly with the increasing levels of resilience**.

**Figure 6:**
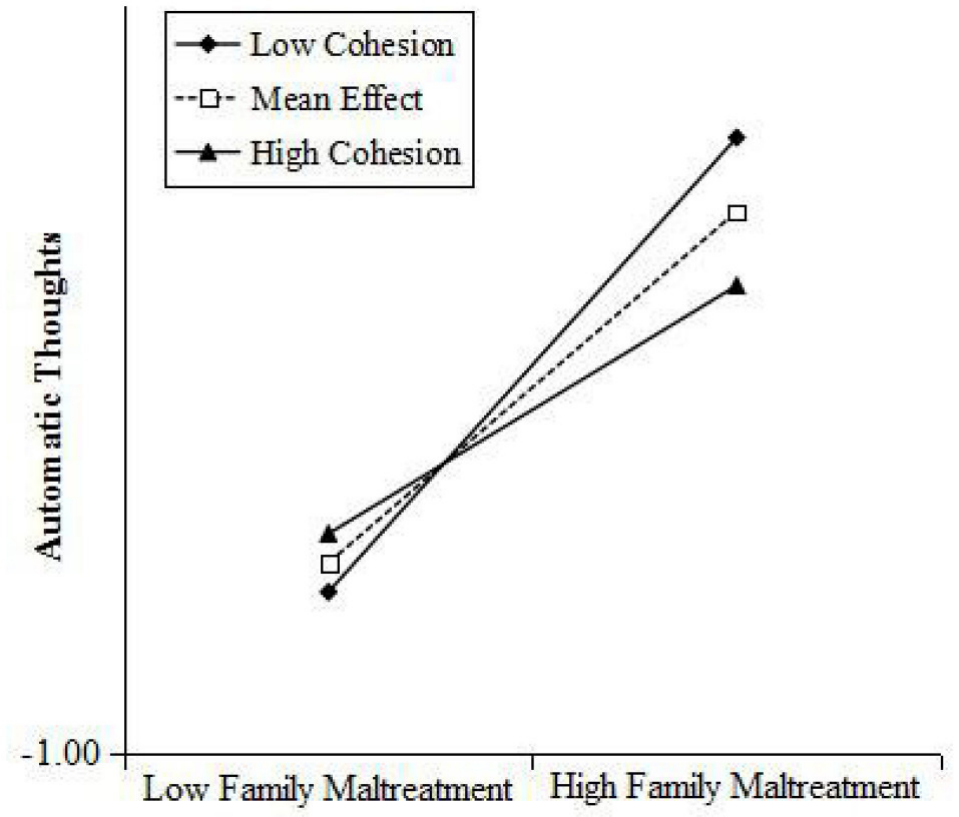
**Cohesion moderated the relationship between family maltreatment and children's automatic thoughts: the risk effects of family maltreatment to their automatic thoughts decreased significantly with the increasing level of family cohesion**.

Simple slope analyses (Aiken and West, 1991) revealed that automatic thoughts increased significantly for individuals who reported lower family cohesion having lower resilience following family maltreatment presented in Figures 5, 6. The risk effects of family maltreatment to children automatic thoughts was negative associated with the increasing levels of resilience. For children with lower resilience, family maltreatment had a positive associated with children's automatic thoughts (*b* = 0.50, *p* < 0.001), and for children with higher resilience, family maltreatment had a positive associated with children's automatic thoughts (*b* = 0.23, *p* < 0.05). The risk effects of family maltreatment to children automatic thoughts was significant negative associated with the increasing level of family cohesion, that with lower family cohesion, family maltreatment had a positive effect on children's automatic thoughts (*b* = 0.61, *p* < 0.001), and with higher cohesion, family maltreatment had a positive effect on children's automatic thoughts (*b* = 0.33, *p* < 0.05).

## Discussion

The current study extended prior understanding of the etiology of child psychopathology by examining familial combined individual effects of both risk and protective factors to the depression among Chinese migrant children with ODD symptoms. Our findings clearly illustrated the pathways that risk factors of both family and individual were positively associated with depression, and individual risk factor mediated the relationship between family risk factor and the depression among migration child with ODD symptoms. Moreover, protective factors moderated the effects of risk factors, that both family cohesion and children's own resilience moderated the relationship between family maltreatment and their automatic thoughts. These findings provided a better understanding for explaining child depression from a family system perspective. And it highlighted the urgent need to prevent family maltreatment and to promote more positive family functioning as well as to focus on children's own characteristics (both risk and protective factors) for migrant children with ODD in China. Additionally, family cohesion, but not adaptability, mitigated the threat of maltreatment to children' automatic thoughts. It seemed that promoting emotional bonding between families was more meaningful than heightening the family ability to the external distress, particularly for relationship-oriented Chinese children.

Risk factors of familial combined with individual were positively associated with children's depression. Consistent to previous findings (Glaser, 2002; Lin et al., 2016), we found that migrant children exposed to family maltreatment were more likely to have high levels of depression. Meanwhile, children with more automatic thoughts had significantly higher levels of depressive symptoms consistent with prior theory (Beck, 1979) and empirically works (Schniering and Rapee, 2002; Clarke and Goosen, 2009). The present study extended previous literature by utilizing a non-Western children sample especially a risk sample of migrant children with ODD symptoms, and confirmed the negative roles of parenting adversity and children's negative cognitive thoughts in their mental healthy across different cultures. While Chinese culture led Chinese parents to incorporate children accomplishments into their views of themselves and Chinese parents feelings of worth were contingent on children performance to a greater extent than both European and African American mothers (e.g., Ng et al., 2014), children with ODD symptoms in China might draw much more parents attention and caused much more family maltreatment.

Importantly, our findings additionally suggested that migrant children's automatic thoughts mediated the relationship between family maltreatment and their depression. As previous literature shown that automatic thoughts were concomitant with a stressful situation leading to depression (Hjemdal et al., 2013; Du et al., 2015), children's negative automatic thoughts were found in current study positively associated with family maltreatment, and further led to their mental health problems. While confirming automatic thoughts as a predictor of depression following the family maltreatment, it is fairly significant to alter migrant children's cognition of interpreting their experience as well as reducing their exposure to maltreatment, particular for the vulnerable migrant children with ODD symptoms.

We also found that protective factors mitigated the threat of risk factors in the moderated mediation model. More specifically, both family cohesion and resilience moderated the effect of family maltreatment on children's automatic. These provided an integrated evidence to the extant research on the respectively buffering effect of family cohesion (Robbins et al., 2003; Xu et al., 2008) and resilience (Wingo et al., 2010; Zhu et al., 2015). Accordingly, protective factors can be viewed as defense mechanisms, which enabled migrant children to thrive in face of adversity and reduced their vulnerability to mental health problems, even the strength of the protective factors were relatively weaker. Here, what was not consistent to our third hypothesis was that the moderated paths existed and only existed in two paths. We did not identify any significant interactions in the directly path between family maltreatment and children's depression, and children's resilience could not moderate the relationship between their automatic thoughts and depression.

For the moderated effects of family functioning, the results from the present study, together with those from other studies (Kashani et al., 1995; Li et al., 2008), demonstrated the outcomes that family cohesion, but not adaptability, mitigated the threat of risk factors to children's depression. While cohesion meant the emotional bonding between family members and adaptability meant the family ability to change in response to the stress, it seemed that the emotional bonding between families was more meaningful than the family ability to the emotional distress, especially for relationship-oriented Chinese (Li et al., 2008). But weather it was similar to the behavior problems still needed more research in the future.

Another important distinction needed to be interpret in the current results was why we did not identify any significant interactions in the directly path between family maltreatment and children's depression, and why children's resilience could not moderate the relationship between their automatic thoughts and depression. As we knew, a high proportion of migrant parents was struggling to cope with the life troubles under a considerable amount of stress and pressure, they may usually adopt simple and crude parenting behavior, neglecting their child's physical and emotional needs (Li et al., 2008, 2016b). Their family maltreatment to migrant children was so severe and the directly pathway to depression was so significant that both family functioning and child individual resilience were unable to buffer against its negative effects. Moreover, migrant children in China used to be left- behind children earlier, so their relationship with their parents was less close (Duan, 2015). Thus, it was unable to resist the strong risk effects of family maltreatment and it can solely moderate partly in the model. With a new perspective on this issue, we apprehended that family maltreatment, to a certain extent, inhibited the ability of family functioning. Additionally, migrant children, particularly with ODD symptom, may have less resilience. Their resilience could only buffer the risk effects against to develop the negative automatic thoughts. And once the negative automatic thoughts formed, their resilience could no longer resist against it.

Findings from our research should be interpreted with caution, given some limitations in this study. First, special participants of migrant children with ODD symptoms recruited in our study, they might have uncommon characters both in the individual and family. Second, our data about all the variables were collected by questionnaire. Although, we considered different reports of parents, teachers and children themselves, there were still some problems interfering with the accuracy of our study. Future studies should include various methods to collect data, such as observational survey and behavioral experiments. Third, in terms of causality, a time line should be implemented while testing the mediation effect of automatic thoughts in relation to depression, longitudinal research is essential in future studies to establish such causal relations.

Despite these limitations, the current study adds to the literature of integrating the potential moderate and mediate factors in a moderated mediation model in a family system perspective. Our findings emphasized the importance to promote more positive family functioning as well as to focus on children's own positive characteristics, and to prevent children' automatic thoughts as well as the family maltreatment for migrant children with ODD in China. Clearly, targeting on reducing the external risk factors (e.g., family maltreatment) may be more effective to promote the mental healthy than appealing to focus on promote the positive ones only.

## Ethics statement

For interested parents of the identified children, psychiatrists from Anding Hospital, mental health counselors, and a family therapist from Center of Family Study and Therapy at Beijing Normal University offered opportunities for ODD treatment. Prior to conducting the study, the Institutional Review Board of Beijing Normal University in China approved the research protocol, including the consent procedure.

## Author contributions

Each of the five authors contribute a lot to the current manuscript. Firstly, XiL and XuL worked out the paper's idea, and discussed the content of the whole manuscript. Secondly, XuL wrote the sections of Introduction, Results, and Discussion. Then, XuL and NZ together conducted the data analysis. Thirdly, XiL and QZ edited the whole paper and revised the language. Finally, YL wrote the part of method. Additionally, DL attended our discussion on manuscript content and gave very useful ideas. Each author read the final version of the current manuscript and support its submission.

## Funding

The study described in this report was Funded by National Social Science Foundation of China, the child development database establishment under rural-to-urban migration context and the establishment of positive youth development system (15ZDB138). The content is solely the responsibility of the authors and does not necessarily represent the official views of State Key Laboratory Foundation, Beijing Organization Committee, and National Social Science Foundation. We are appreciative of the parents, children, and teachers who participated in our study and the many people who assisted in data collection.

### Conflict of interest statement

The authors declare that the research was conducted in the absence of any commercial or financial relationships that could be construed as a potential conflict of interest.
